# Cationic dye removal (crystal violet) using *Equisetum palustre*

**DOI:** 10.1039/d6ra04824b

**Published:** 2026-07-22

**Authors:** Niyazi Erdem Delikanlı, Baybars Ali Fil, Betül Tuba Gemici, Handan Ucun Özel, Halil Barış Özel

**Affiliations:** a Bartin University, Faculty of Engineering, Architecture and Design, Department of Environmental Engineering 74100 Bartin Turkey btakcay@bartin.edu.tr; b Balikesir University, Faculty of Engineering, Department of Environmental Engineering Balikesir Turkey; c Bartin University, Faculty of Forestry, Department of Silviculture 74100 Bartin Turkey

## Abstract

In this study, the removal of cationic dyes from synthetic wastewater by batch-mode biosorption processes was investigated using *Equisetum palustre* as a biosorbent. The adsorption capacity of the biosorbent was investigated under the influence of parameters such as adsorbent dose, initial dye concentration, pH and temperature. The isotherm fitting of the experimental data was investigated using isotherm models such as Langmuir, Freundlich, Temkin, Sips, Toth and Khan. The adsorption kinetic behavior of the biosorbent was evaluated using pseudo-first-order, pseudo-second-order and Elovich kinetic models. The obtained experimental results fitted well with the Sips isotherm model and the pseudo-second-order kinetic model. Furthermore, thermodynamic parameters were studied, and the activation energy (*E*_a_) was determined to be 11.142 kJ mol^−1^ for an adsorbent dosage of 10 g L^−1^, a shaking speed of 150 rpm, an initial dye concentration of 100 mg L^−1^ and a pH of 6.2. The enthalpy change (ΔH*) was 8.621 kJ mol^−1^, whereas the entropy change (ΔS*) was −0.249 kJ mol^−1^. The results revealed that *Equisetum palustre* (EP) might be exploited as an alternative to conventional biosorbents in wastewater treatment for the elimination of dyes.

## Abstract 2

In this study, the removal of cationic dyes from synthetic wastewater by batch-mode biosorption processes was investigated using *Equisetum palustre* as a biosorbent.

## Graphical abstract

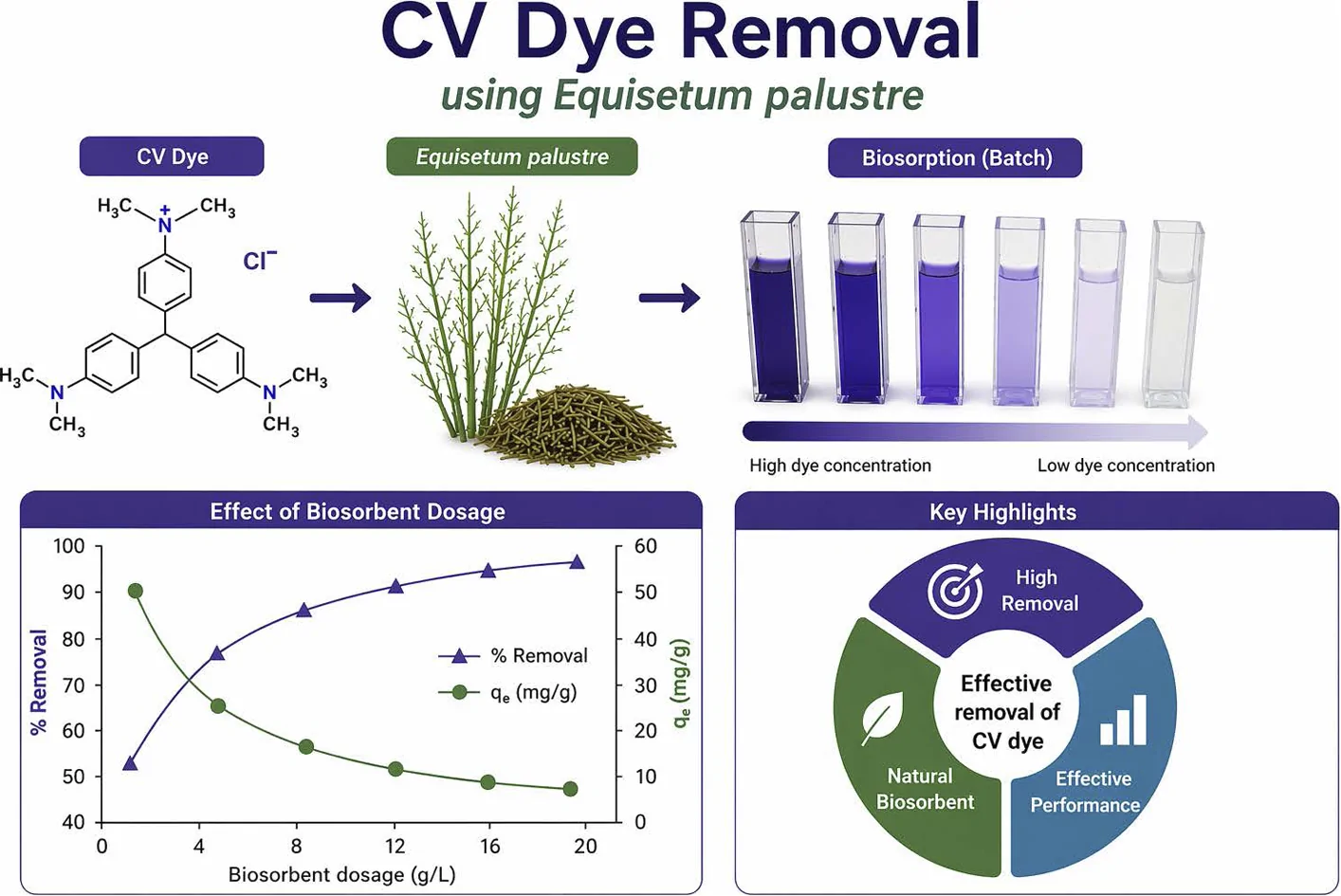

## 1. Introduction

Among the most serious kinds of water pollutants are dye effluents from the manufacturing and use of dyes in the agricultural, pulp, polymer, and cotton sectors, among others. Moreover, the biological load is enhanced when pollutants are discharged into freshwater environments because most dyes are not biodegradable.^1^ Because some of these effluents have carcinogenic effects on living species, their release into various environments poses serious health concerns.^2^ Water coloration in rivers and streams creates a barrier for gas exchange and sunlight transmission, thereby inhibiting photosynthesis. When employed for irrigation, the toxic qualities of colored effluents destroy microbial activity, thereby affecting crop yields.^3^

Crystal violet (CV), which is classified as a triarylmethane dye, is also a basic dye. CV is substantially used in human and veterinary medicine, biological staining applications, and textile coloration. Because of its antimicrobial activities, CV is widely used in chicken feed to prevent the development of fungus. Although it has practical applications, CV has been categorized as a hazardous substance due to its mutagenic and cancer-causing effects. It can disrupt cellular division and injure the eyes, notably the retina and mucosa, upon exposure for longer periods. As a consequence, it is vital to apply correct treatment techniques to inhibit the spread of CV across ecosystems.

The remediation of dye wastewater using coagulation/flocculation,^4^ electrocoagulation,^5^ filtration,^6^ adsorption,^7^ ozonation,^8^ and sedimentation^9^ has thus been the focus of research. Biosorption methods have the advantages of being economical and straightforward when compared to their alternative methods.

For increased economic possibilities and decreased environmental issues, biosorption has been adopted as an innovative method for treating wastewater.^10^ Different adsorbents, such as biomass residues, microalgae, fungi, and bacterial cultures, have been employed for the sorption of dyes.

The utilization of low-cost, sustainable, and easily accessible adsorbent materials, including peat,^11^ coconut husk,^12,13^ chitosan,^14^ palm tree fiber,^15^ kudzu,^16^ fly ash,^17^ and clay,^18^ has become a major research focus in the development of cost-effective, non-toxic and surface-engineered adsorbents.

The goal of this research was to study the CV sorption process from the aqueous phase onto *Equisetum palustre* (EP) to evaluate *Equisetum palustre*'s ability as a biosorbent. For this purpose, numerous variables such as pH, initial dye concentration, temperature, adsorbent dosage, and reaction time were examined. The surface characteristics of EP were evaluated using FTIR spectroscopy and BET analysis in order to identify the functional groups involved in CV adsorption and assess the surface area and pore volume of the biosorbent. The first section was aimed at investigating the adsorption mechanism between CV and *Equisetum palustre* by examining the validity of various isotherm and kinetic models. The Langmuir, Freundlich, and Temkin equations were commonly employed for two-parameter models, while the Toth, Sips, and Khan equations were commonly utilized for three-parameter models. In order to examine the biosorption process, the pseudo-first-order, pseudo-second-order, and Elovich equations kinetic models were employed. Calculations were performed for the thermodynamic parameters *E*_a_, ΔG*, ΔH*, and ΔS*.

## 2. Materials and methods

### 2.1. Preparation of adsorbents

*Equisetum palustre* was collected from a local source in Bartin, Turkey. To remove impurities, *Equisetum palustre* was cleaned many times in purified water after being dried in a microwave at 36 °C for 4 hours. The sorbent was air-dried in the sun for one day and then ground to a size of 0.5–1 mm. After drying the samples in an oven at 80 °C for 24 hours, the experiments were performed.

### 2.2. Chemicals

**Fig. 1:**
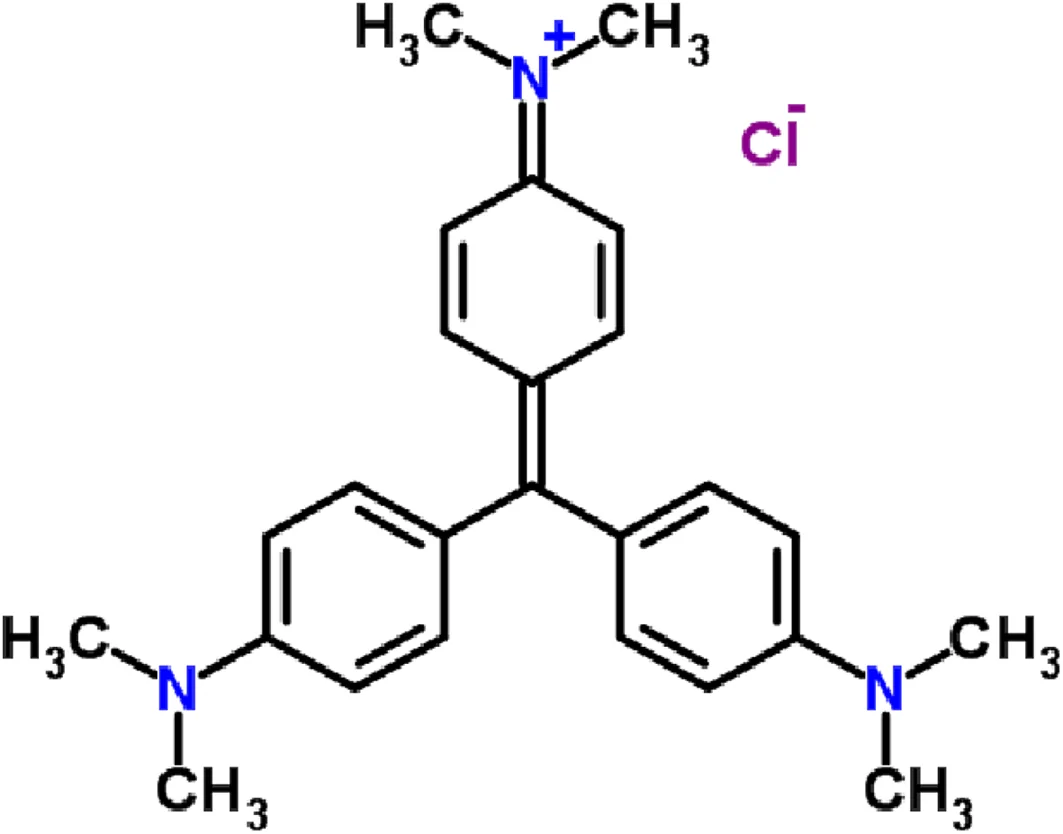
Structure of crystal violet.

All reagents used were of analytical grade. Water used to make all the solutions was distilled twice. Crystal violet (CV), also known as tris(4-(dimethylamino)phenyl)methylium chloride, is a monovalent cationic primary dye with a molecular weight of about 407.98 g mol^−1^, represented as CI 42555. It was purchased from Merck (Turkey). Its chemical formula is C_25_H_30_ClN_3_.^19^ The structure of the CV dye is shown in Fig. 1. The stock solution was diluted with purified water to obtain different working concentrations needed for the experiments. To change the pH, solutions of HCl and NaOH were utilized.

### 2.3. Experimental procedure

In the batch studies, the effects of the biosorbent dose (1–20 g L^−1^), initial pH (2–9), temperature (15–45 °C), and contact duration were examined. The solutions were mixed in a Jeio Tech IST-4075R orbital shaker (150 rpm) at 25 °C. In a typical batch experiment, 1 g of the adsorbent was added to 100 mL of the crystal violet solution at a concentration of 100 mg L^−1^. The sediments and solutions from the samples were separated using a centrifuge (Nüve NF200, Ankara, Turkey) at 5000 rpm for 5 min. Crystal violet dye concentrations were measured at 586 nm using a UV-Vis spectrophotometer (Hach Lange DR6000, Düsseldorf, Germany).^20–22^ All experiments in this research were performed in triplicate.

The concentrations of the solutions before and after dye adsorption were used to calculate the quantity of dye adsorbed.^23^

The following formula was used to determine the adsorbed dye concentration *q*_e_:1
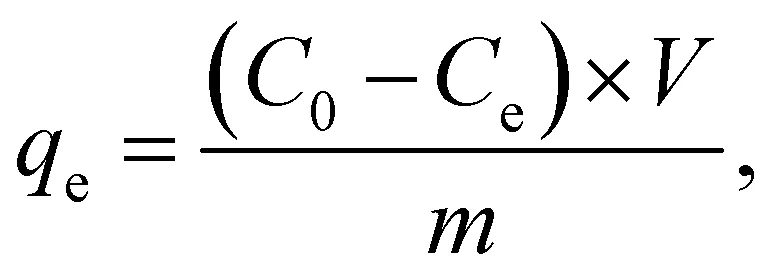
where *q*_e_ (mg g^−1^) is the amount of dye adsorbed, *C*_0_ (mg L^−1^) is the initial dye concentration, *C*_e_ (mg L^−1^) is the concentration of dye in the solution at equilibrium, *V* (L) is the volume, and *m* (g) is the amount of the adsorbent used.

The following formula was used to determine the adsorbed dye concentration *q*_*t*_:2
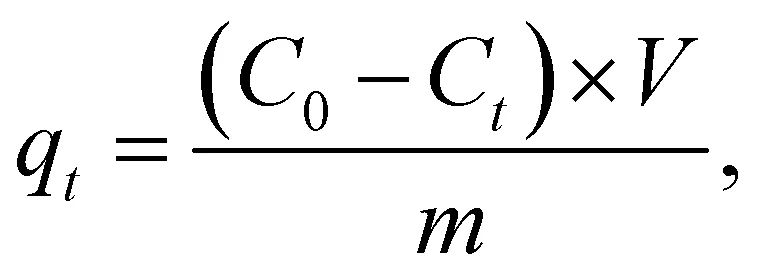
where *q*_*t*_ (mg g^−1^) is the amount of dye adsorbed, *C*_0_ (mg L^−1^) and *C*_*t*_ (mg L^−1^) are the initial dye concentration and the concentration at time *t*, respectively. *V* is the volume of the solution (L), and *m* is the mass of the biosorbent (g).

### 2.4. Properties of *Equisetum palustre*

Fourier-transform infrared (FTIR) spectroscopy analysis was carried out using a Shimadzu IRAffinity-1 spectrometer in the wavenumber range of 4000–700 cm^−1^ to identify the surface functional groups of EP before and after CV adsorption. In addition, the specific surface area and pore volume of the EP biosorbent were determined by nitrogen adsorption–desorption measurements using the Brunauer–Emmett–Teller (BET) method with a Quantachrome Autosorb iQ Station 1 system.

## 3. Result and discussion

### 3.1. Characterization of *Equisetum palustre*

**Fig. 2:**
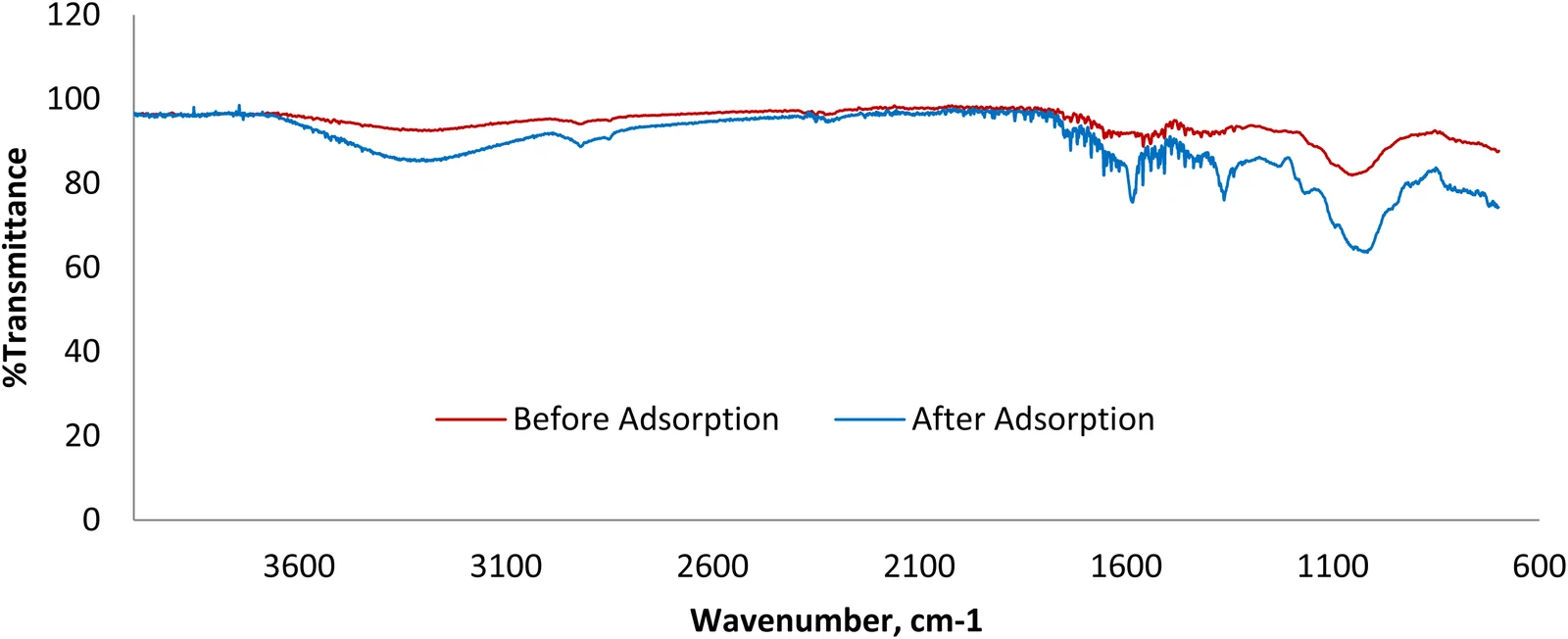
FTIR spectra of EP before and after biosorption of CV.

The FTIR spectra of EP were recorded before and after CV biosorption in order to identify the functional groups involved in the adsorption process (Fig. 2). Before biosorption, the characteristic absorption bands were observed at 1031, 2845, 2918, and 3302 cm^−1^. The band at 1031 cm^−1^ can be attributed to the C–O and C–C stretching vibrations of the carbohydrate structures, while the bands at 2845 and 2918 cm^−1^ are associated with the C–H stretching vibrations of the CH_3_ and CH_2_ groups, respectively. The broad band observed at 3302 cm^−1^ indicates the hydrogen-bonded O–H stretching vibrations related to the alcohol and phenolic groups. The comparison of the FTIR spectra of EP before and after CV adsorption in Fig. 2 shows that no remarkable structural change occurred after the adsorption process. The band observed at around 1584 cm^−1^ may be attributed to aromatic C=C stretching vibrations associated with the lignin and phenolic structures in the plant-based biosorbent. In addition, the band appearing at 1362 cm^−1^ after biosorption may be assigned to C–H bending vibrations.

The porous characteristics of the EP biomass were evaluated by nitrogen adsorption–desorption analysis using the Brunauer–Emmett–Teller (BET) approach. The material exhibited a specific surface area of 1.778 m^2^ g^−1^ and a total pore volume of 6.423 × 10^−3^ cm^3^ g^−1^. The BET surface area of EP is comparable to the values previously reported for untreated agricultural and lignocellulosic biosorbents, which generally range from 1.38 to 11.2 m^2^ g^−1^.^24^ The biosorption of CV onto EP is likely not controlled solely by its porous structure but also by the contribution of surface functional groups and adsorbent–dye interactions.

### 3.2. Effect of initial dye concentration on biosorption

**Fig. 3:**
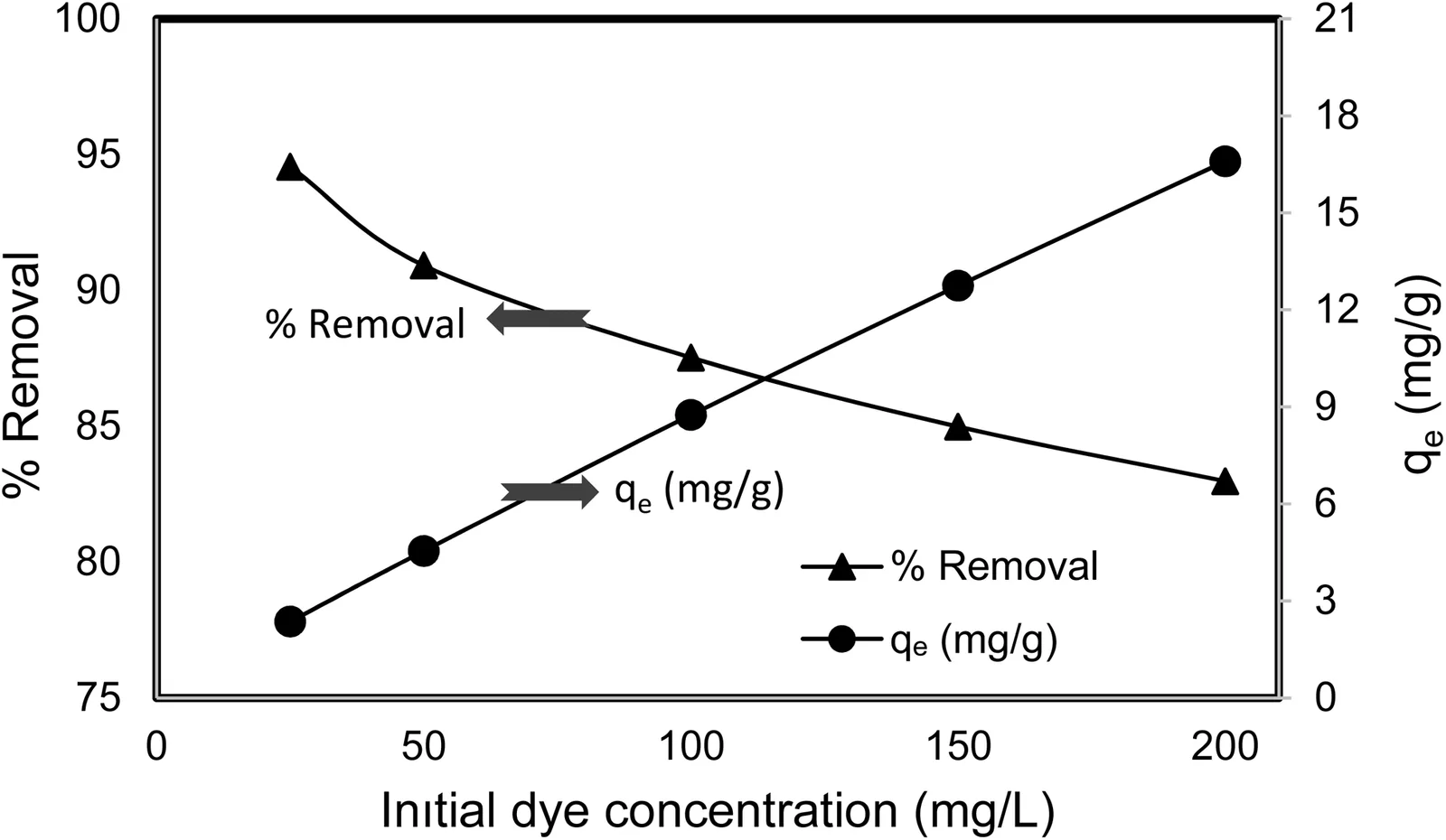
Effect of initial dye concentration on biosorption of CV (pH: 6.2, *T*: 25 °C, AD: 10 g L^−1^, and SS: 150 rpm).

For studying the effects of initial dye concentration on crystal violet removal, initial dye concentrations between 25 and 200 mg L^−1^ were chosen. The experimental conditions included room temperature (25 °C), natural pH of the effluent (6.2), a biosorbent dosage of 10 g L^−1^, a shaking speed of 150 rpm and a reaction time of 240 min. The experimental results obtained are shown in Fig. 3. As can be seen from the results, there was a significant decrease in the calculated removal efficiency as the amount of dye increased. The separation efficiency for the initial dye content of 25 mg L^−1^ was calculated to be 94.56%, while the separation efficiency at the initial dye content of 200 mg L^−1^ remained at 82.98%. Although the removal efficiency declined, the amount of dye adsorbed per unit of biosorbent increased significantly. We can see this trend from the amount of dye removed per unit of biosorbent. The *q*_e_ values for 25, 50, 100, 150 and 200 mg L^−1^ were 2.36, 4.55, 8.75, 12.75 and 16.60 mg g^−1^, respectively. Increasing the dye concentration was the driving force for binding to the active sites on the biosorbent surface. This concentration gradient also increased the amount of dye adsorbed.^25^

### 3.3. Effect of pH on biosorption

**Fig. 4:**
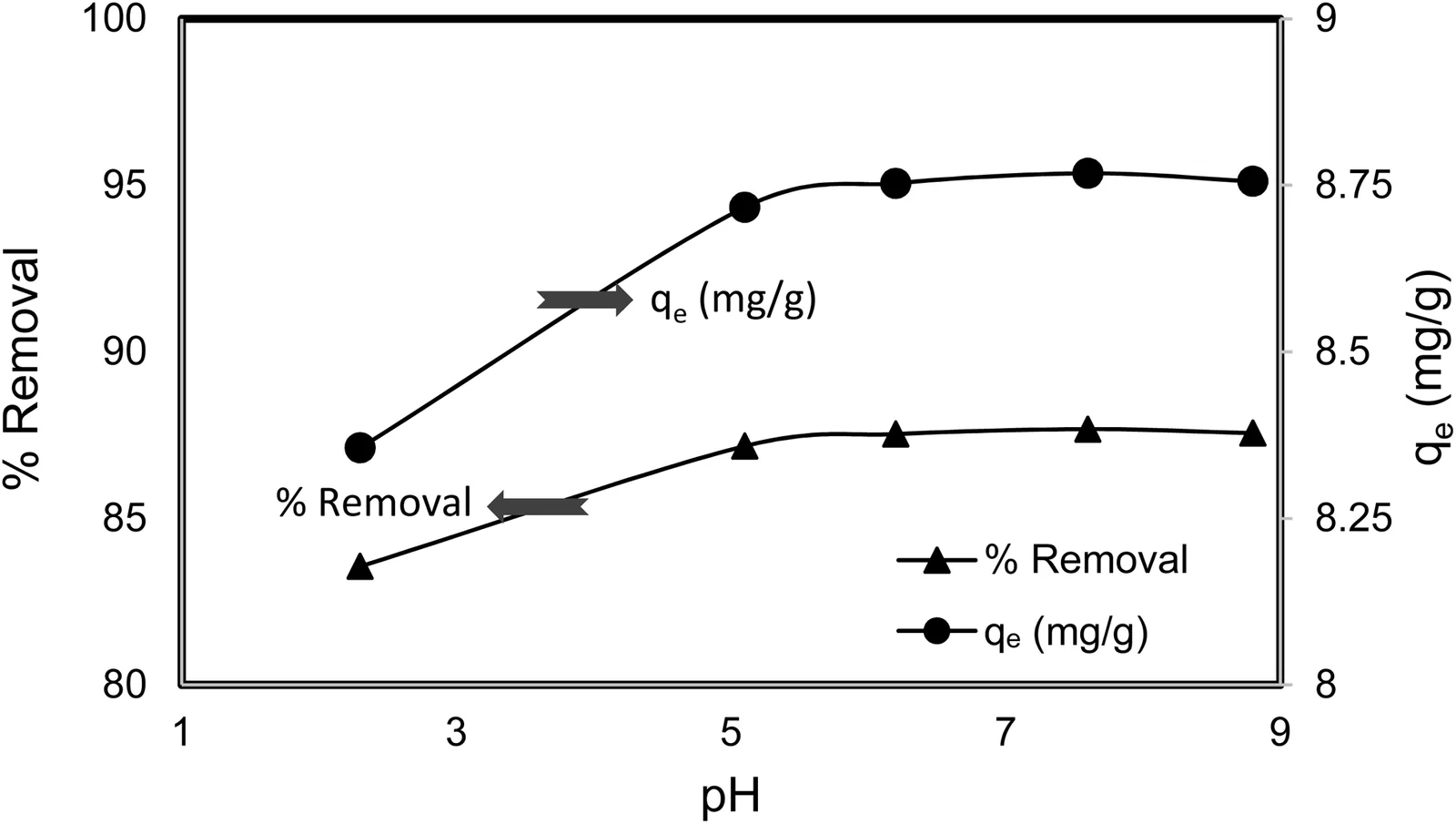
Effect of pH values on biosorption of CV (*C*_0_: 100 mg L^−1^, *T*: 25 °C, AD: 10 g L^−1^, and SS: 150 rpm).

The surface adsorption equilibrium of CV on EP was investigated at 25 °C, an adsorbent dosage of 10 g L^−1^, and a shaking speed of 150 rpm. This study lasted 240 minutes at 100 mg per L initial dye concentration. The effect of pH on the rate at which CV is removed from EP can be seen in Fig. 4. The amount of CV that EP could adsorb increased with an increase in pH. The pH_iep_ value of EP can be used to explain this phenomenon. The number of negatively charged adsorbent sites decreased and the number of positively charged surface sites increased when the pH of the system reduced below the pH_iep_ value of the adsorbent, which did not promote the adsorption of cationic dyes due to electrostatic repulsion. Positively charged dye cations can bind better to negatively charged adsorption sites at higher pH values due to electrostatic attractive forces.^26^

### 3.4. Effect of adsorbent dosage on biosorption

**Fig. 5:**
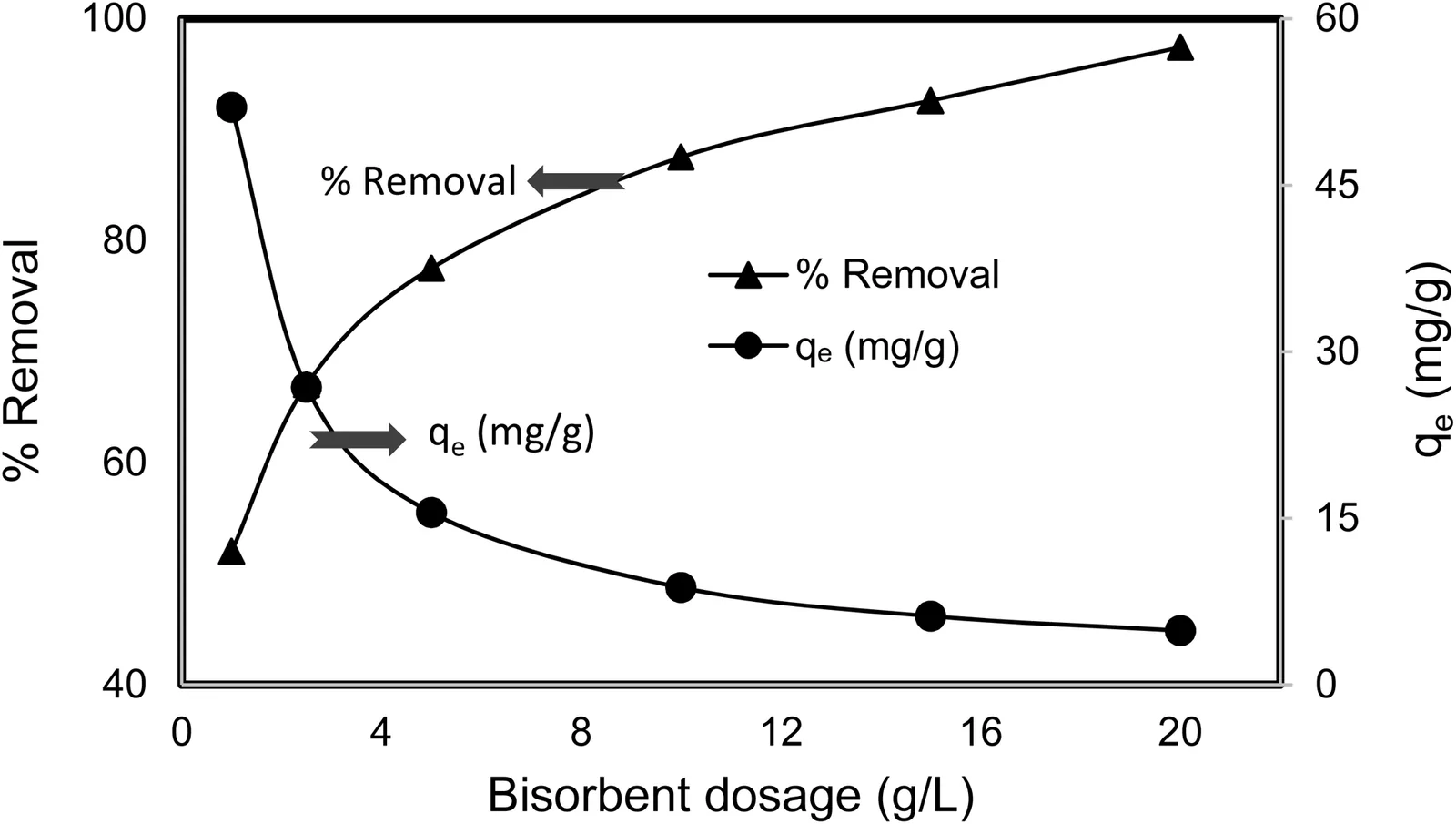
Effect of adsorbent dosage on biosorption (*C*_0_: 100 mg L^−1^, pH: 6.2, *T*: 25 °C, and SS: 150 rpm).

Fig. 5 shows the results of the adsorption experiment when the biosorbent dosage is changed from 1 to 20 g L^−1^ while maintaining a constant CV concentration (100 mg L^−1^). Although the adsorption capacity of the adsorbent decreased from 1 to 20 g L^−1^ as the amount of the biosorbent increased, the proportion of CV sorption increased. This could be explained by the fact that more adsorption sites are available because there is more sorbent. When the sorbent-to-solute concentration ratio is smaller, very rapid sorption occurs on the sorbent surface, resulting in a lower concentration of solute in the solution.^27^

### 3.5. Effect of temperature on biosorption

**Fig. 6:**
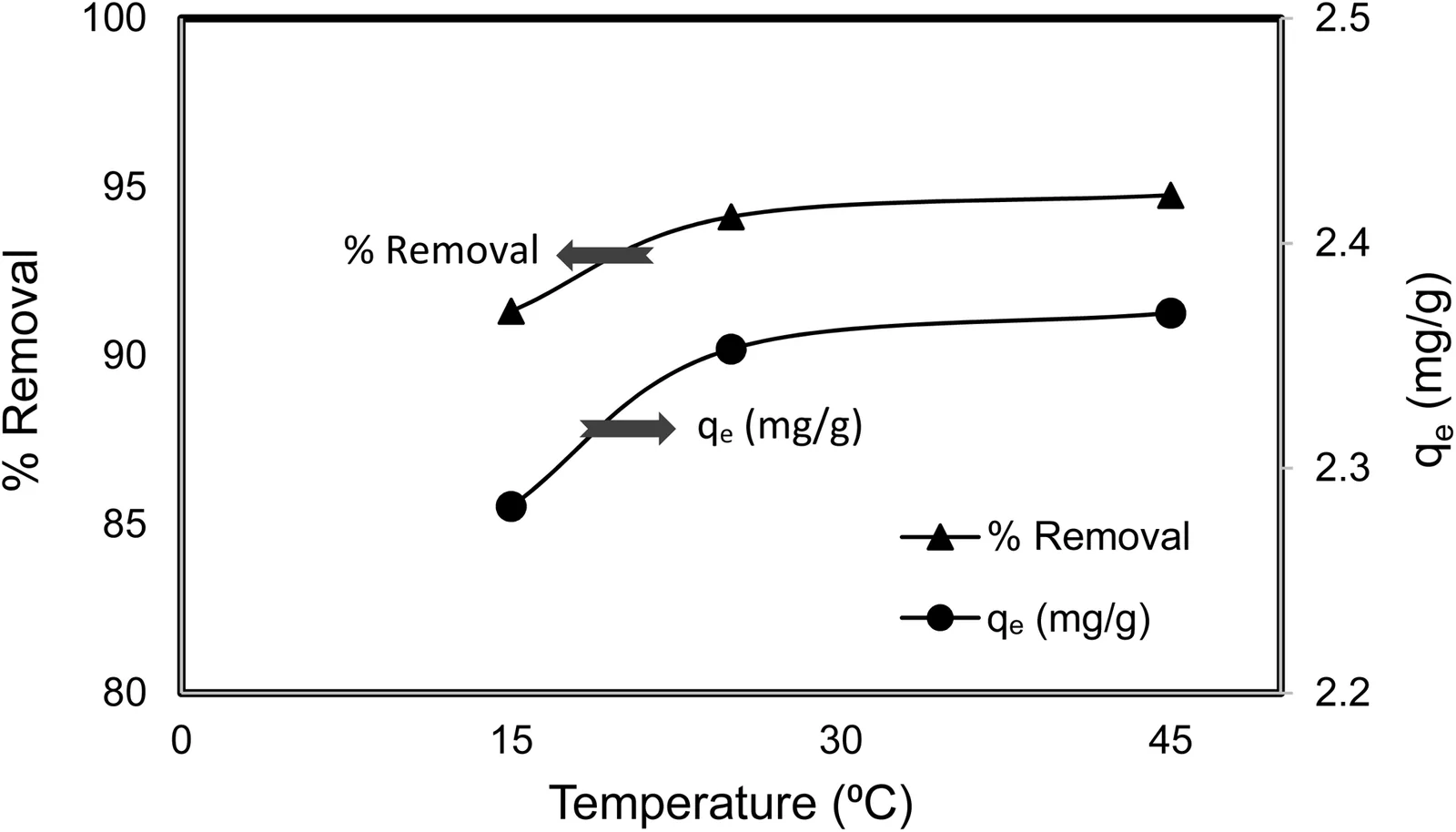
Effect of temperature on biosorption (*C*_0_: 100 mg L^−1^, pH: 6.2, AD: 10 g L^−1^, and SS: 150 rpm).

It has long been recognized that temperature affects the adsorption process in two main ways: due to the decrease in solution viscosity caused by an increase in temperature, the rate of diffusion of the adsorbate molecules across the outer boundary layer and inside the adsorbent particles increases. In addition, a change in temperature alters the equilibrium capacity of the adsorbent for a specific adsorbate. The influence of temperature on the dynamic adsorption of CV on EP is shown in Fig. 6. It is shown that the increase in dye adsorption on EP increases with temperature, indicating that the process of dye adsorption is endothermic. The rate of CV adsorption appears to be significantly affected by temperature.^28^

### 3.6. Effect of contact time on biosorption

**Fig. 7:**
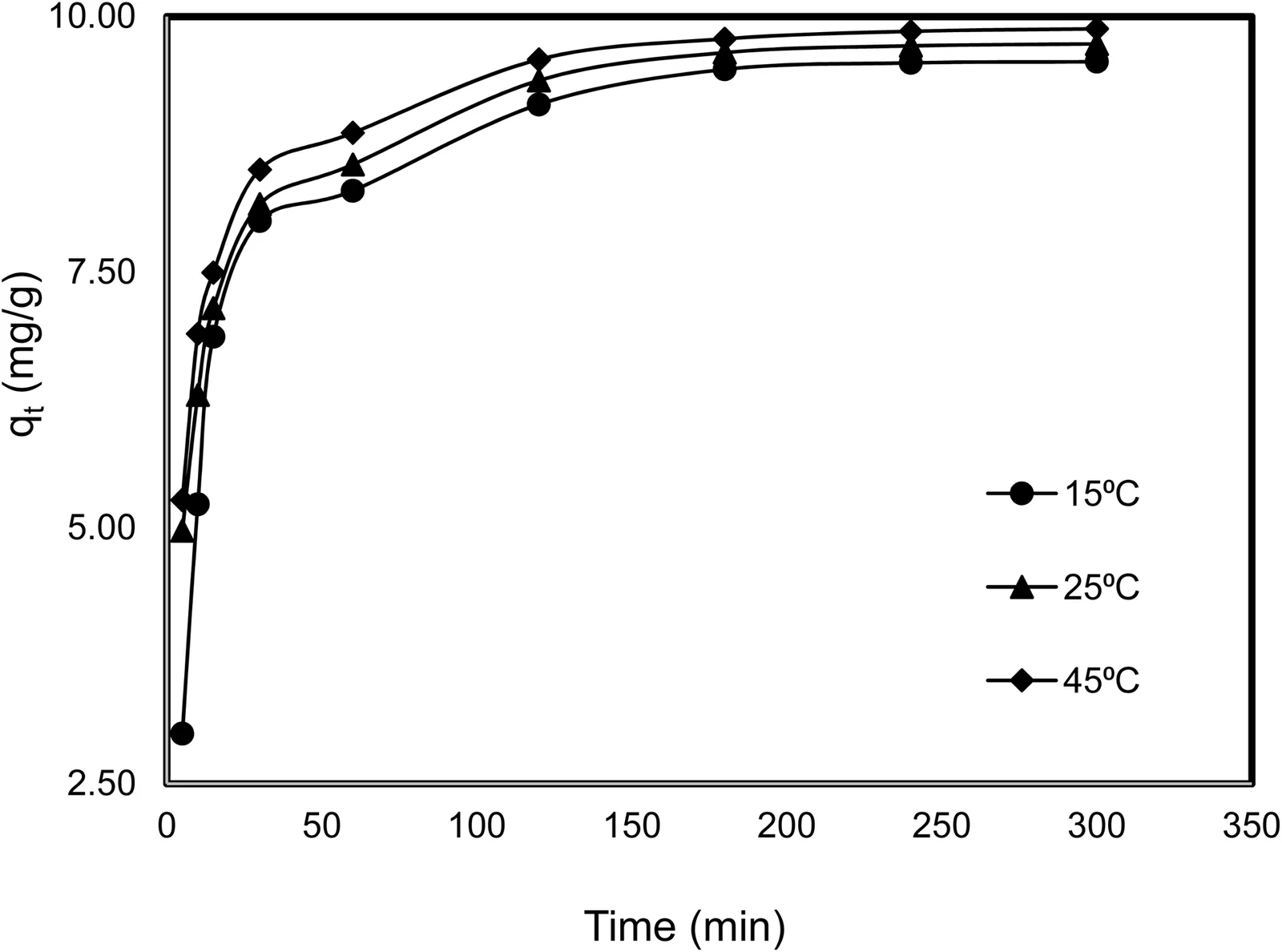
Effect of contact time on biosorption (*C*_0_: 100 mg L^−1^, pH: 6.2, AD: 10 g L^−1^, and SS: 150 rpm).

Fig. 7 shows the results of the analysis of CV dye adsorption onto a 10 g per L dosage of EP at pH = 6.2 at different temperatures (15, 25 and 45 °C) and time intervals. Independent of the temperature of the suspension, an initially high and rapid rate of CV adsorption was observed over a period between 0 and 10 min. The reason for a larger concentration gradient and higher diffusion rate of CV dye to a solid surface was that the adsorbent initially contained the greatest number of activation sites for adsorbing CV dye either on the surface or at the interlayer site. Due to the decreasing CV concentration gradient, a reduction in active adsorbent sites, and a declining diffusion rate to the solid surface, the CV dye adsorption rate followed significantly slower kinetics after 10 min under all conditions. Finally, in all cases, the adsorption capacity was saturated, but at higher temperatures, such as 45 °C, this occurred significantly earlier at 60 min compared to 180 min at 15 °C and 25 °C.^29^

### 3.7. Adsorption isotherms

From a theoretical perspective, the classical density functional theory (DFT) can provide a more fundamental description of ion adsorption isotherms by considering molecular-level interactions and density distributions near adsorbent surfaces.^30,31^ However, DFT-based approaches generally require more detailed structural information and involve a relatively high computational burden. Therefore, within the experimental scope of the present study, the appropriateness of the equilibrium data from the experiments was evaluated using the Langmuir, Freundlich, Temkin, Sips, Toth, and Khan equations. The mathematical formulas for these models are presented in Table 1.^32–37^

Isotherm model equations (a) and kinetic model equations (b)

**Table:** 

	Isotherm	Mathematical equations	Eqn	References
(a)	Langmuir	*q*_e_ = (*q*_m_ × *K*_L_ × *C*_e_)/(1 + *K*_L_ × *C*_e_)	(3)	32
	Freundlich	*q*_e_ = *K*_F_ × *C*_e_^1/*n*^	(4)	33
	Temkin	*q*_e_ = (*R* × *T*/*b*) × ln(*K*_T_ × *C*_e_)	(5)	34
	Sips	*q*_e_ = (*q*_m_ × *a*_S_ × *C*_e_^1/*n*^)/(1 + *a*_S_ × *C*_e_^1/*n*^)	(6)	35
	Toth	*q*_e_ = (*q*_m_ × *C*_e_)/(*K*_To_ + *C*_e_^*n*^)^1/*n*^	(7)	36
	Khan	*q*_e_ = (*q*_m_ × *b*_K_ × *C*_e_)/(1 + *b*_K_ × *C*_e_)^*a*_K_^	(8)	37

**Table:** 

	Kinetic model	Mathematical equations	Eqn	References
(b)	Pseudo-first-order rate model	ln(*q*_e_ − *q*_*t*_) = ln *q*_e_ − *k*_1_*t*	(9)	38
	Pseudo-second-order rate model	*t*/*q*_*t*_ = [1/*k*_2_*q*_e_^2^] + (1/*q*_e_)*t*	(10)	39
	Elovich model	*q*_*t*_ = *β* ln(*αβ*) + *β* ln *t*	(11)	40

**Fig. 8:**
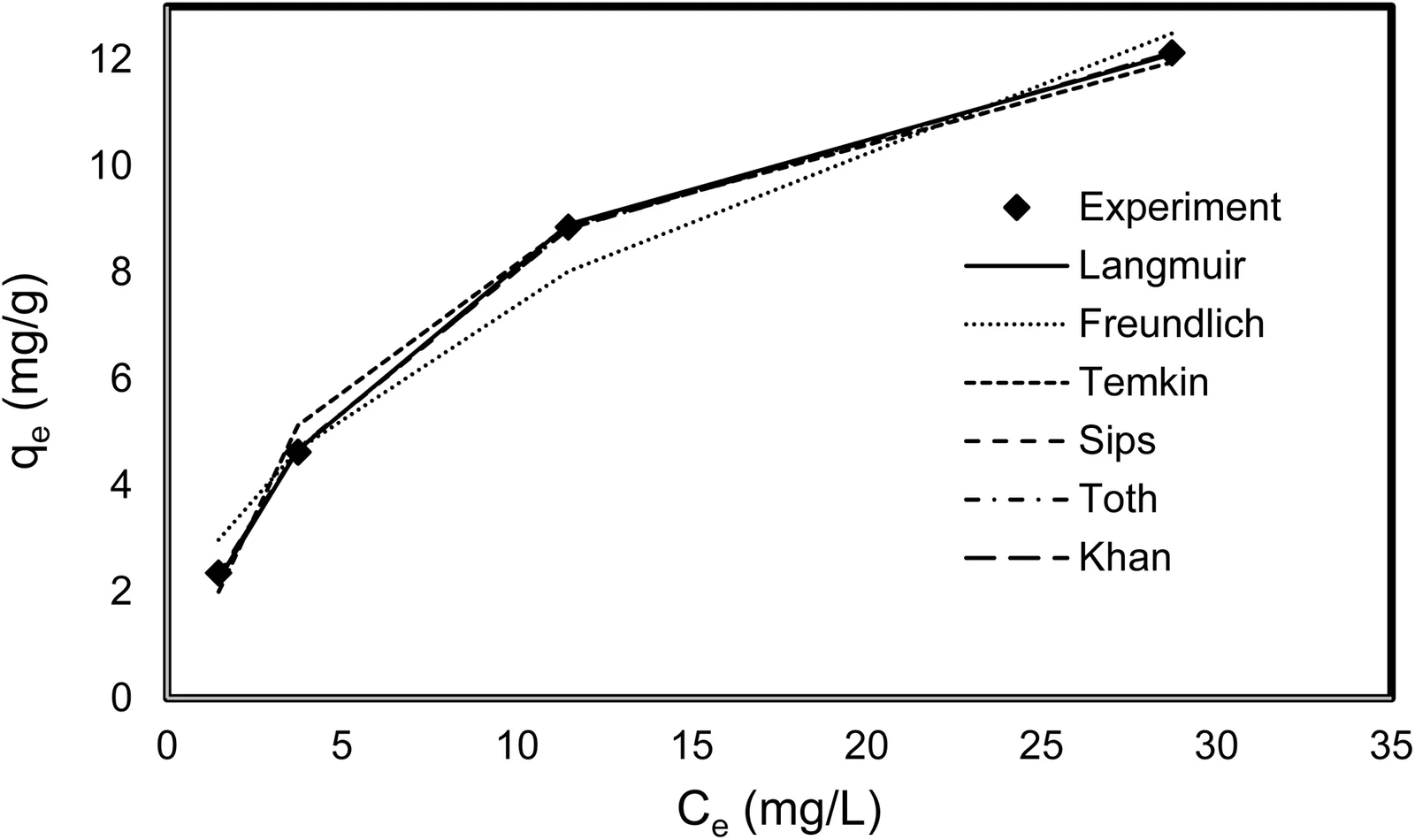
Comparison of isotherm models for biosorption (*C*_0_: 100 mg L^−1^, pH: 6.2, AD: 10 g L^−1^, *T*: 25 °C, and SS: 150 rpm).

In both systems (batch and fixed bed), the amount of dye molecules adsorbed per mass unit of biosorbent (*q*_eq_) increases rapidly with increasing initial concentration of the adsorbate solution. The biosorption is dependent on the initial concentration because at a low initial solution concentration, fewer adsorption sites are accessible due to a lower ion concentration and limited availability of target ions.^41^ The result is that as the ion concentration increases, biosorption increases. With increasing ion concentrations, every gram of the biosorbent is accessible to more ions, and these ions might behave in different ways depending on the system. The *R*^2^ values of the Sips and Langmuir models were found to be greater than those of others listed in Table 2. The fitted curves are presented as non-linear plots in Fig. 8 and also support these findings. The adsorption capacity of EP was compared with other plant-based and lignocellulosic adsorbents used for CV removal, and the results are presented in Table 3.

**Table 2:** Isotherm constants for CV biosorption onto *Equisetum palustre*

Temperature		288 K	298 K	318 K
Langmuir	*q*_m_	15.9311	15.8947	17.6672
	*K*_L_	0.0768	0.1110	0.1152
	*R* ^2^	0.99970	0.99983	0.99995
Freundlich	*n*	1.9463	2.0728	1.9389
	*K*_F_	1.9375	2.4728	2.6285
	*R* ^2^	0.98369	0.98928	0.98982
Temkin	*b*	6.9431	7.298013	7.0238
	*K*_T_	0.8306	1.2363	1.2922
	*R* ^2^	0.99751	0.99286	0.99569
Sips	*q*_m_	16.9372	16.6608	17.7403
	*a*_S_	0.0779	0.1121	0.1153
	*n*	1.0629	1.0576	1.0049
	*R* ^2^	0.99984	0.99996	0.99995
Toth	*q*_m_	8.6221	11.4546	18.6869
	*K*_To_	9.0249	7.3037	8.9486
	*n*	1.1639	1.0861	0.9863
	*R* ^2^	0.99991	0.99991	0.99995
Khan	*q*_m_	11.7524	13.4100	18.1269
	*b*_K_	0.1108	0.1369	0.1118
	*a*_K_	0.8592	0.9207	1.0139
	*R* ^2^	0.99991	0.99982	0.99995

**Table 3:** Comparison of the CV uptake capacities of different adsorbents from the literature

Adsorbents	*q*_max_ (mg g^−1^)	References
*Platanus orientalis* leaf	25.88	42
Pineapple leaves	9.78	43
Anatolian black pine	12.36	20
*Moringa oleifera* pod husk	156.25	24
Raw *Eucalyptus* leaves	88.81	44
*Arundo donax* L.	19.60	45
Cedar cone	13.646	46
*Equisetum palustre*	15.89	This study (25 °C)

### 3.8. Adsorption kinetics

**Fig. 9:**
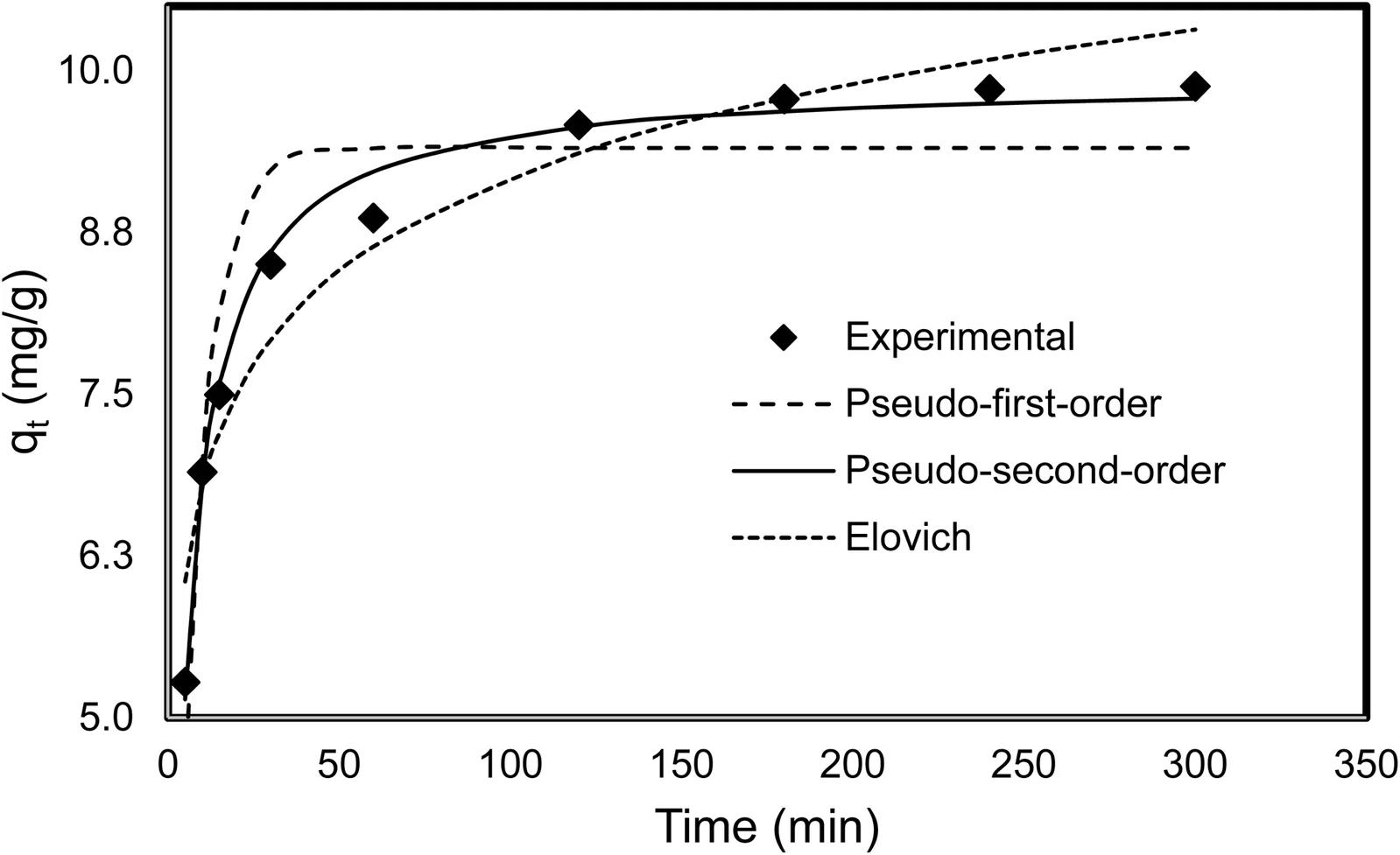
Comparison of kinetic models for biosorption (*C*_0_: 100 mg L^−1^, pH: 6.2, AD: 10 g L^−1^, *T*: 25 °C, and SS: 150 rpm).

It is widely known that diffusional barrier is crucial to total solute transport, and that sorbent surface characteristics have a major influence on sorption rate parameters. To understand how the sorption of the examined ions evolves over time, many kinetic models were tested. The Elovich model, pseudo-first-order, and pseudo-second-order rate models were used to derive the rate constant of the removal of CV using *Equisetum palustre* from solution. The mathematical equations of these models are given in Table 1.^38–40^ According to the coefficient of correlation *R*^2^, the pseudo-first-order model and the pseudo-second-order model matched the experimental data somewhat better than the Elovich model. To put it another way, the pseudo-second-order model is better at capturing CV adsorption kinetics. Many biosorption systems' kinetics have been effectively characterized using this paradigm.^47^ Although the pseudo-second-order model fitted the kinetic data well, this finding alone should not be regarded as direct evidence of chemisorption. The model may indicate that the adsorption rate is associated with the availability of adsorption sites and the interaction between CV molecules and the EP surface. Therefore, to obtain a more balanced interpretation, the adsorption mechanism was also evaluated using the activation thermodynamic parameters. The positive ΔG* values at 288, 298, and 318 K indicate the presence of an activation barrier during CV adsorption onto EP. In addition, the relatively low ΔH* value suggests that strong chemical bond formation may not be the only factor controlling the adsorption process. Table 4 contains the calculated *k*_2_ (g mg^−1^ min^−1^) and *R*^2^ values. Fig. 9 shows the results recorded for the initial CV concentrations.

**Table 4:** Kinetic constants for CV biosorption onto *Equisetum palustre*

Temperature		288 K	298 K	318 K
Pseudo-first-order	*q*_e_	9.1924	9.2270	9.4036
	*k*_1_	0.0825	0.1185	0.1335
	*R* ^2^	0.9929	0.9833	0.9863
Pseudo-second-order	*q*_e_	9.8708	9.7800	9.9368
	*k*_2_	0.0118	0.0190	0.0216
	*R* ^2^	0.9947	0.9980	0.9988
Elovich	*β*	0.6948	0.8932	0.9593
	*α*	5.8849	33.0257	69.1595
	*R* ^2^	0.9742	0.9931	0.9918

### 3.9. Activation parameters and thermodynamic parameters

**Fig. 10:**
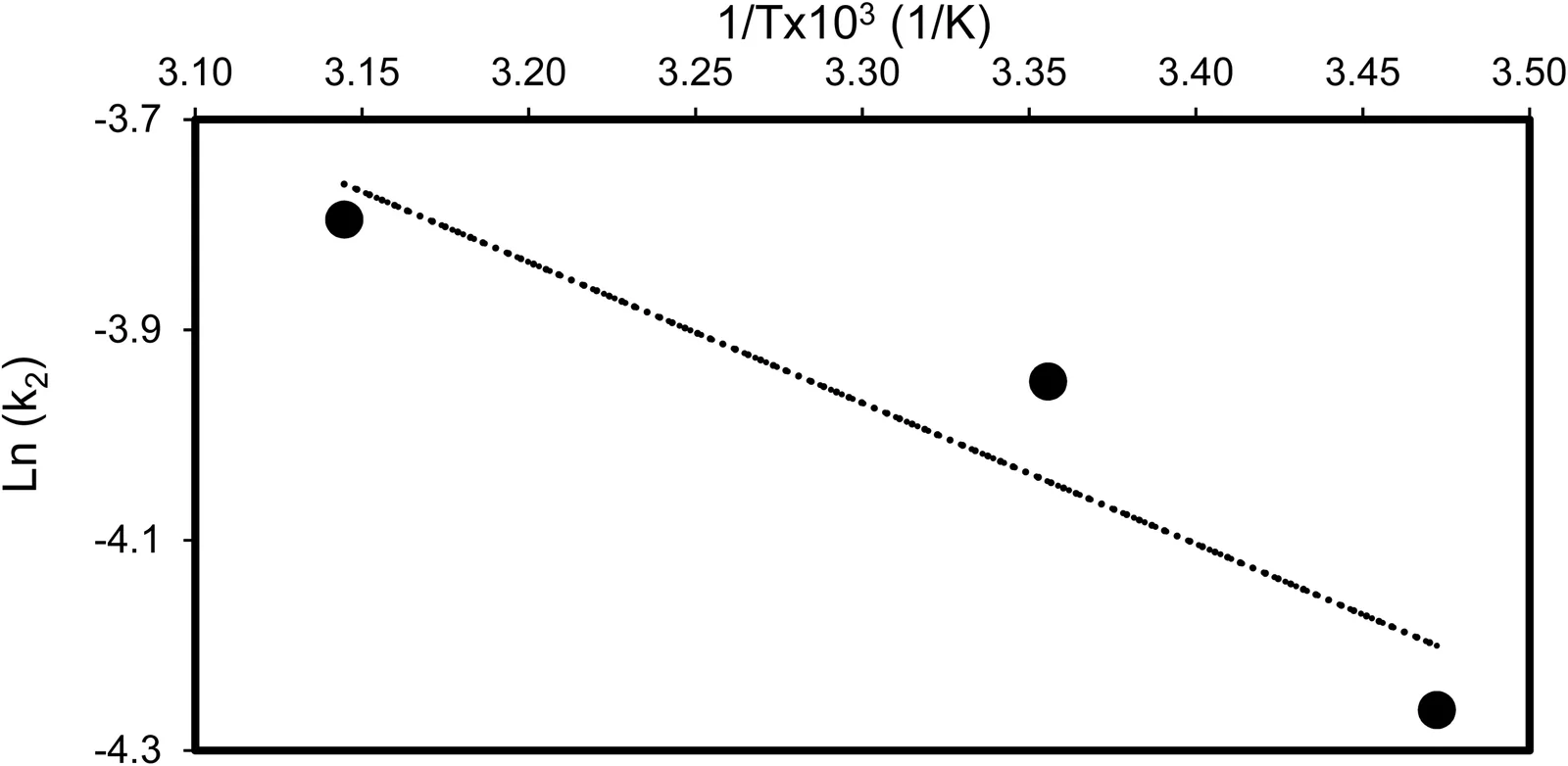
Arrhenius plots for the biosorption of the dye on *Equisetum palustre*.

The activation energy of CV adsorption on *Equisetum palustre* (EP) was calculated from the second-order rate constants reported in Table 4 using the Arrhenius equation:12
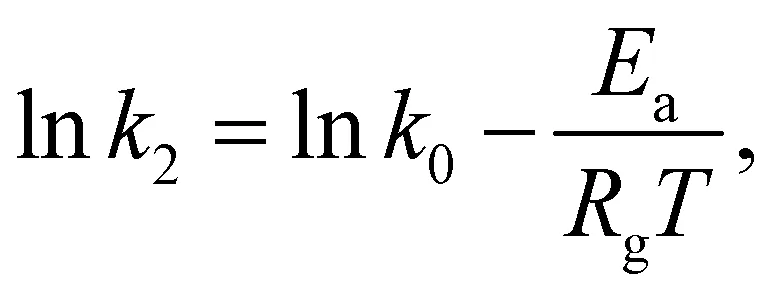
where *E*_a_ is the activation energy (kJ mol^−1^); *k*_2_ is the rate constant of sorption (g mg^−1^ min^−1^); *k*_0_ is the Arrhenius factor, which is the temperature-independent factor (g mg^−1^ min^−1^); *R*_g_ is the gas constant (J mol^−1^ K^−1^); and *T* is the solution temperature (K). The slope of the plot of ln *k*_2_*versus* 1/*T* is used to evaluate *E*_a_, which was found to be 11.142 kJ mol^−1^ for CV adsorption (Fig. 10).

**Table 5:** Thermodynamic parameters of CV biosorption onto *Equisetum palustre*

	*T* (K)		
	288	298	318
ΔG* (kJ mol^−1^)	80.5086	83.0047	87.9968
ΔH* (kJ mol^−1^)		8.6219	
ΔS* (kJ mol^−1^ K^−1^)		−0.2496	

The free energy (ΔG*), enthalpy (ΔH*) and entropy (ΔS*) values of activation can be calculated using the Eyring equation:^48^13
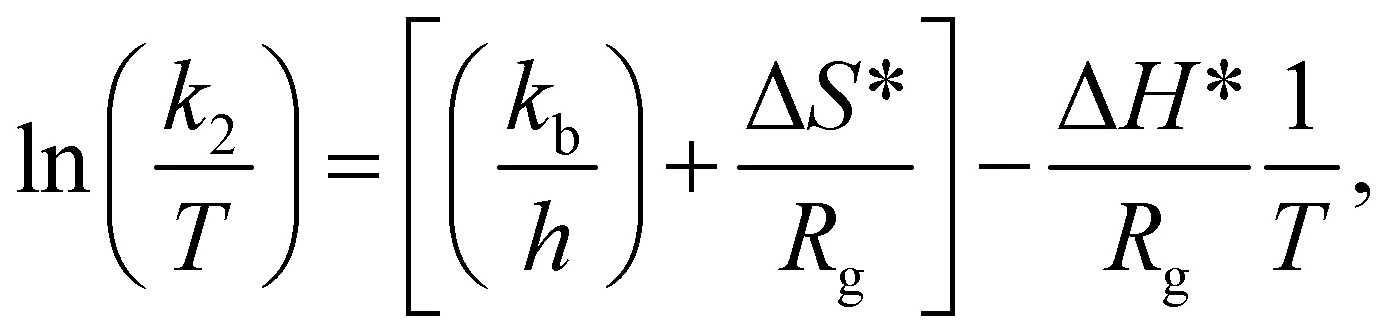
where *k*_b_ and *h* are the Boltzmann and Planck's constants, respectively. According to eqn (13), a plot of ln(*k*_2_/*T*) *versus* 1/*T* should be a straight line with a slope −ΔH*/*R*_g_ and an intercept of (ln(*k*_b_/*h*) + ΔS*/*R*_g_). ΔH* and ΔS* were calculated from the slope and intercept of the line, respectively. The Gibbs energy of activation can be written in terms of the entropy and enthalpy of activation as follows:

14 ΔG* = ΔH* − *T* × ΔS*.

**Fig. 11:**
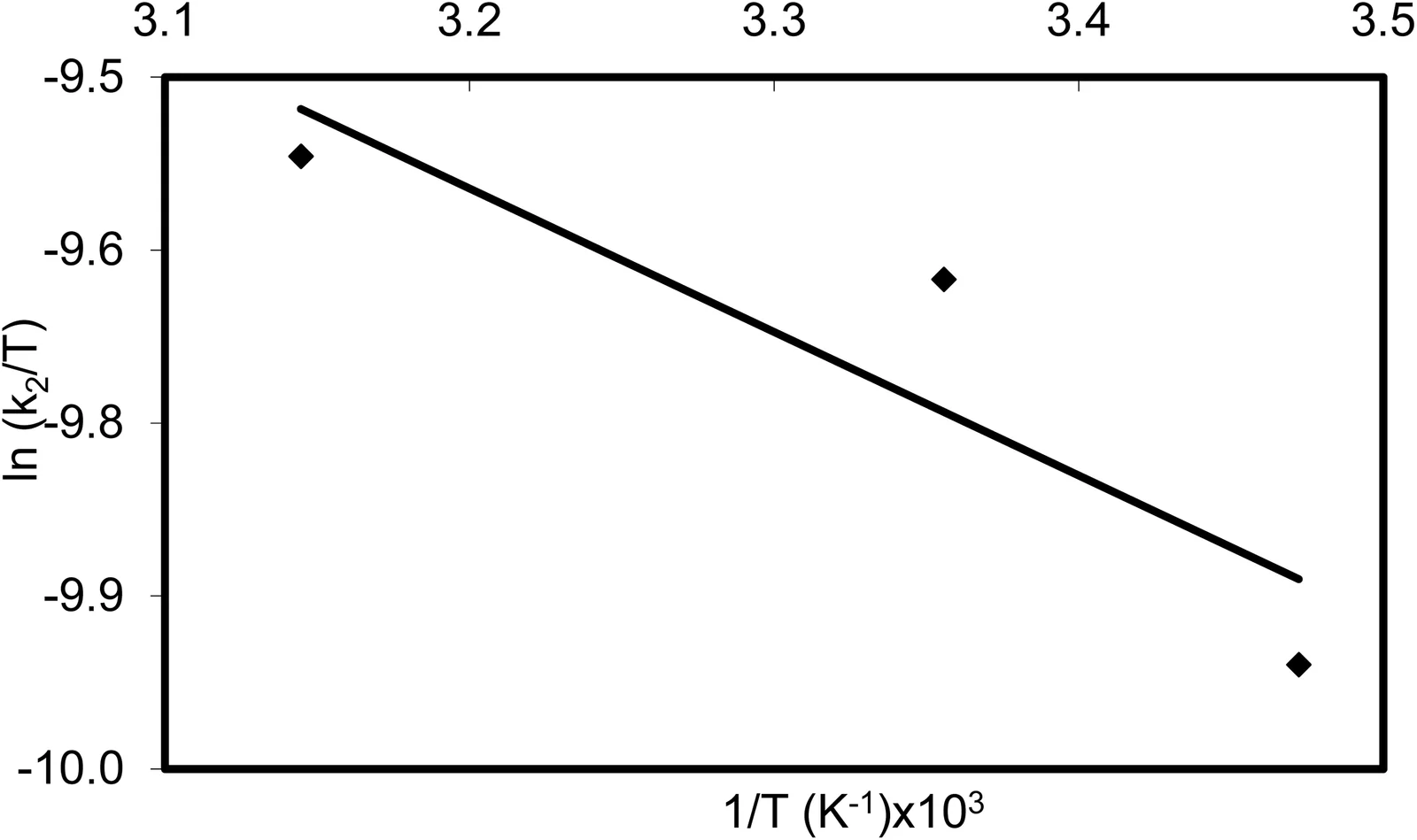
Plots of ln(*k*_2_/*T*) *versus* 1/*T* for the biosorption of the dye on *Equisetum palustre*.

It is found that the values of the free energy (ΔG*), enthalpy (ΔH*) and entropy (ΔS*) of activation for CV are 83.005 kJ mol^−1^, 8.621 kJ mol^−1^ and −0.249 kJ mol^−1^ K^−1^, respectively, at 298 K (Fig. 11). The results are shown in Table 5.

The positive ΔG* values indicate that an activation energy barrier exists during the adsorption of CV onto EP. Therefore, these values may be interpreted as the energy requirement for the formation of the activated complex. Additionally, the increase in ΔG* with temperature suggests that the energy requirement for reaching the activated state becomes higher at elevated temperatures. The positive ΔH* value indicates that the activation step of CV adsorption has an endothermic character. Moreover, the relatively low ΔH* value suggests that the adsorption process should not be attributed solely to strong chemical bond formation. The negative ΔS* value indicates a decrease in randomness at the solid-solution interface during the transition state, which may be related to a more ordered arrangement of CV molecules near the biosorbent surface.

### 3.10. Standard deviation analysis

Standard deviation (SD) was used as an additional statistical criterion to evaluate the agreement between the experimental data and the values predicted by the adsorption isotherm and kinetic models. Lower SD values generally indicate a closer agreement between the experimental and calculated adsorption capacities and may reflect a better model fit. The standard deviation was calculated using eqn (15):15
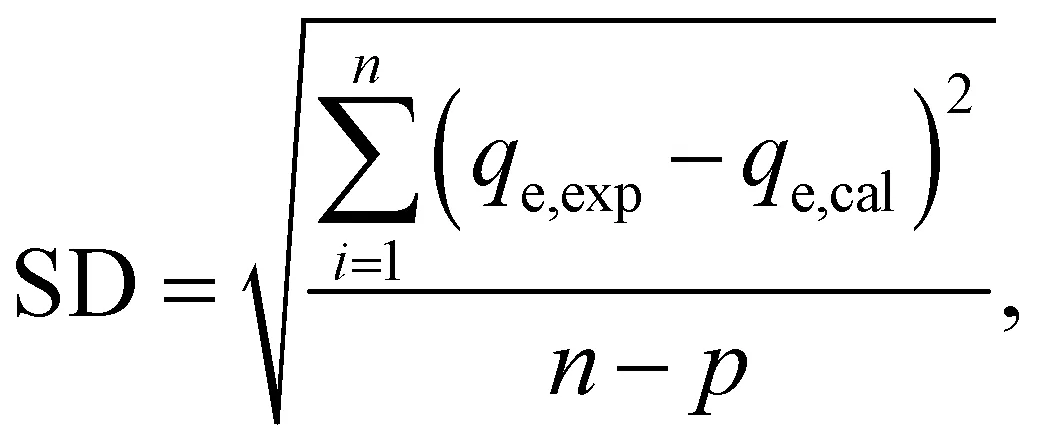
where *q*_e,exp_ and *q*_e,cal_ are the experimental and calculated adsorption capacities (mg g^−1^), respectively, *n* is the number of experimental observations, and *p* is the number of parameters in the corresponding model. In this study, the standard deviation values obtained for both the adsorption isotherm and kinetic models were compared together with the coefficient of determination (*R*^2^) to identify the model that best described the experimental data. Models exhibiting higher *R*^2^ values and lower SD values were considered to provide the most reliable representation of the biosorption process.

#### 3.10.1. Standard deviation analysis of the isotherm models

Standard deviation (SD) was employed as a statistical indicator to evaluate the agreement between the experimental adsorption capacities and those predicted by the Langmuir isotherm model. Lower SD values generally indicate a better agreement between the experimental and calculated equilibrium adsorption capacities. As presented in Table 6, the Langmuir model yielded SD values of 4.156, 4.391, and 4.627 at 15, 25, and 45 °C, respectively. The gradual increase in SD values with increasing temperature suggests a slight increase in the deviation between the experimental and predicted adsorption capacities. Nevertheless, the differences remained relatively small, indicating that the Langmuir model maintained a satisfactory fitting performance over the investigated temperature range. The relatively low SD values, together with the high coefficient of determination (*R*^2^), demonstrate that the Langmuir isotherm adequately describes the equilibrium biosorption behavior of crystal violet onto *Equisetum palustre*. These findings imply that the adsorption process can be reasonably represented by monolayer adsorption occurring on a homogeneous surface with a finite number of energetically equivalent adsorption sites.

**Table 6:** Statistical parameters of the experimental and model-predicted *q*_e_ values for the isotherm models at different temperatures

Temperature	Model	Mean	Variance	Standard deviation
15 °C	*q*_e_ Experiment	6.712	17.126	4.138
	*q*_e_ Langmuir	6.703	17.269	4.156
	*q*_e_ Freundlich	6.761	15.983	3.998
	*q*_e_ Temkin	6.711	17.067	4.131
	*q*_e_ Sips	6.715	17.058	4.130
	*q*_e_ Toth	6.716	17.038	4.128
	*q*_e_ Khan	6.716	17.038	4.128
25 °C	*q*_e_ Experiment	6.990	18.987	4.357
	*q*_e_ Langmuir	6.974	19.280	4.391
	*q*_e_ Freundlich	7.042	17.601	4.195
	*q*_e_ Temkin	6.990	18.855	4.342
	*q*_e_ Sips	6.988	19.016	4.361
	*q*_e_ Toth	6.984	19.100	4.370
	*q*_e_ Khan	6.994	19.052	4.365
45 °C	*q*_e_ Experiment	7.221	21.334	4.619
	*q*_e_ Langmuir	7.217	21.406	4.627
	*q*_e_ Freundlich	7.282	19.745	4.443
	*q*_e_ Temkin	7.221	21.151	4.599
	*q*_e_ Sips	7.219	21.373	4.623
	*q*_e_ Toth	7.216	21.431	4.629
	*q*_e_ Khan	7.216	21.431	4.629

#### 3.10.2. Standard deviation analysis of the kinetic models

SD was employed to evaluate the agreement between the experimental adsorption capacities and those predicted by the kinetic models (Table 7). Lower SD values indicate a better agreement between the experimental and calculated values, and therefore a more accurate description of the adsorption kinetics.

**Table 7:** Statistical parameters of the experimental and model-predicted *q*_*t*_ values for the kinetic models at different temperatures

Temperature	Model	Mean	Variance	Standard deviation
15 °C	*q*_*t*_ Experiment	7.681	5.190	2.278
	*q*_*t*_ Pseudo-first-order	7.680	5.043	2.246
	*q*_*t*_ Pseudo-second-order	7.702	4.660	2.159
	*q*_*t*_ Elovich	8.241	7.857	2.803
25 °C	*q*_*t*_ Experiment	8.179	2.919	1.709
	*q*_*t*_ Pseudo-first-order	8.143	3.240	1.800
	*q*_*t*_ Pseudo-second-order	8.173	3.007	1.734
	*q*_*t*_ Elovich	8.180	2.768	1.664
45 °C	*q*_*t*_ Experiment	8.460	2.581	1.607
	*q*_*t*_ Pseudo-first-order	8.432	2.818	1.679
	*q*_*t*_ Pseudo-second-order	8.457	2.667	1.633
	*q*_*t*_ Elovich	8.460	2.403	1.550

The SD values obtained for the pseudo-second-order model were 2.159, 1.734, and 1.633 at 15, 25, and 45 °C, respectively. Compared with the pseudo-first-order model, the pseudo-second-order model exhibited consistently lower standard deviation values over the investigated temperature range. Moreover, the SD values gradually decreased with increasing temperature, suggesting that the predictive capability of the pseudo-second-order model improved under higher-temperature conditions.

The relatively low SD values indicate that the pseudo-second-order model provides excellent agreement between the experimental and calculated adsorption capacities throughout the biosorption process. When considered together with the high coefficient of determination (*R*^2^), these results indicate that the pseudo-second-order model is the most appropriate kinetic model for describing the biosorption of crystal violet onto *Equisetum palustre*. The superior fitting performance of this model suggests that it can reliably represent the overall adsorption kinetics under the investigated experimental conditions.

### 3.11. Practical applicability and future studies

Although the present study was conducted using synthetic crystal violet solutions, evaluating EP under real wastewater conditions would provide further insights into its practical applicability. In such systems, common inorganic ions such as Na^+^ and NH_4_^+^, as well as other organic constituents, may coexist with dye molecules and influence the adsorption behavior. Therefore, future studies may further examine the adsorption performance and selectivity of EP under multi-component conditions.

From a practical perspective, the recovery and reusability of EP after CV adsorption are also important issues. One possible strategy for improving solid–liquid separation is the magnetic modification of EP with Fe_3_O_4_ nanoparticles. Magnetic adsorbents can be readily separated from aqueous media using an external magnetic field, reducing the need for additional filtration or centrifugation steps. Fe_3_O_4_-based magnetic modification has been reported as an effective strategy for facilitating the recovery and reuse of adsorbents in repeated adsorption cycles.^49^ Accordingly, future studies may focus on developing Fe_3_O_4_-modified EP composites and evaluating their adsorption–desorption performance, selectivity, and recyclability under more realistic wastewater conditions.

## 4. Conclusions

In this study, dye removal was successfully performed with *Equisetum palustr*e from an aqueous solution containing crystal violet, a cationic dye. In this way, an attempt was made to purify the dyes contained in wastewater from the textile industry using the natural biosorbent *Equisetum palustre* and to measure its adsorption capacity for the dyes. The test results showed the best agreement with the Sips model among all the isotherm models, and it was observed that *Equisetum palustre* effectively adsorbed the dye. The positive enthalpy value indicated that the system followed an endothermic process, and the negative entropy value indicated that the system disorder decreased during the adsorption process.

## Author contributions

Niyazi Erdem Delikanli: conceptualization, investigation, data curation, validation, visualization. Baybars Ali Fil: methodology, formal analysis, data curation, writing – original draft. Betul Tuba Gemici: data curation, validation, visualization, writing – review and editing. Handan Ucun Ozel: conceptualization, supervision, validation, writing – review and editing. Halil Baris Ozel: conceptualization, resources, supervision, writing – review and editing.

## Conflicts of interest

The authors have no conflicts of interest to declare that are relevant to the content of this article.

## Data Availability

The data supporting the findings of this study are included within the article. No additional datasets were generated or analysed beyond those presented in the manuscript.
